# Triple-Negative Breast Cancer and Predictive Markers of Response to Neoadjuvant Chemotherapy: A Systematic Review

**DOI:** 10.3390/ijms24032969

**Published:** 2023-02-03

**Authors:** Nadine S. van den Ende, Anh H. Nguyen, Agnes Jager, Marleen Kok, Reno Debets, Carolien H. M. van Deurzen

**Affiliations:** 1 Department of Pathology, Erasmus MC Cancer Institute, Erasmus University Medical Centre, 3015 GD Rotterdam, The Netherlands; 2 Department of Medical Oncology, Erasmus MC Cancer Institute, Erasmus University Medical Centre, 3015 GD Rotterdam, The Netherlands; 3 Department of Medical Oncology, Tumor Biology & Immunology, Netherlands Cancer Institute, 1066 CX Amsterdam, The Netherlands

**Keywords:** TNBC, breast cancer, NAC, TILs, Ki-67, prediction, pCR

## Abstract

Around 40–50% of all triple-negative breast cancer (TNBC) patients achieve a pathological complete response (pCR) after treatment with neoadjuvant chemotherapy (NAC). The identification of biomarkers predicting the response to NAC could be helpful for personalized treatment. This systematic review provides an overview of putative biomarkers at baseline that are predictive for a pCR following NAC. Embase, Medline and Web of Science were searched for articles published between January 2010 and August 2022. The articles had to meet the following criteria: patients with primary invasive TNBC without distant metastases and patients must have received NAC. In total, 2045 articles were screened by two reviewers resulting in the inclusion of 92 articles. Overall, the most frequently reported biomarkers associated with a pCR were a high expression of Ki-67, an expression of PD-L1 and the abundance of tumor-infiltrating lymphocytes, particularly CD8+ T cells, and corresponding immune gene signatures. In addition, our review reveals proteomic, genomic and transcriptomic markers that relate to cancer cells, the tumor microenvironment and the peripheral blood, which also affect chemo-sensitivity. We conclude that a prediction model based on a combination of tumor and immune markers is likely to better stratify TNBC patients with respect to NAC response.

## 1. Introduction

Breast cancer is one of the most common malignancies among women worldwide [1]. Up to 15% of all breast cancers are triple-negative [2]. Triple-negative breast cancer (TNBC) is defined by the lack of expression of the estrogen receptor (ER), the progesterone receptor (PR) and the absence of human epidermal growth factor receptor 2 (HER2) overexpression and/or gene amplification. According to the guidelines of the American Society of Clinical Oncology/College of American Pathologists (ASCO/CAP), ER and PR are considered negative when <1% of tumor cells show nuclear staining via immunohistochemistry [3,4]. TNBC is a biological and clinically heterogeneous disease and it tends to be more common among younger women and women carrying a *BRCA1* gene mutation. Over the last decades, several gene-expression-based classifications have emerged for TNBC [5,6,7]. The majority of TNBC cases, as determined by immunohistochemistry, cluster within the basal-like intrinsic subtype, but a small group is identified as non-basal-like, including the luminal androgen receptor subtype and that which is HER2-enriched.

Patients with TNBC are not eligible for endocrine or HER2-targeted therapies, currently rendering chemotherapy as the most-used therapeutic option [8]. Anthracycline/taxane-containing chemotherapy regimens are widely used as adjuvant or neoadjuvant chemotherapy (NAC) and approximately 40 to 50% of patients with TNBC treated with chemotherapy achieve a pathologic complete response (pCR) [9,10,11,12]. Platinum salts can be added to taxane-containing neoadjuvant treatment, for example, carboplatin in combination with paclitaxel [13,14]. Currently, neoadjuvant treatment is the preferred treatment choice, based on the prognostic value of the treatment response and the possibility to treat the non-pCR group with novel promising adjuvant therapies. Achieving a pCR is correlated with a good prognosis, equal to that of other breast cancer subtypes, and is considered a surrogate marker for survival [15]. Unfortunately, there is also a subgroup of patients with TNBC that experience no or limited benefit from chemotherapy [8]. This chemo-resistant subgroup shows highly aggressive behavior with high recurrence rates, an increased risk of metastases and a lack of recognized molecular targets for therapy [16,17,18]. Moreover, these patients endure multiple cycles of chemotherapy with hardly any benefit, indicating the need for predictive biomarkers to prevent unnecessary toxicity and costs.

Many researchers have already investigated whether standard clinicopathologic characteristics at baseline, including tumor size, histologic grade, histologic subtype and lymph node involvement, can predict the response to NAC, albeit with contradictory results. For example, some articles found an association between the smaller, lower-grade, non-metastasized tumors (N0) and pCR rate, whereas other articles reported no association, indicating that these characteristics cannot be solely used for the prediction of a pCR [19,20,21,22,23,24,25,26]. Regarding histologic subtype, no significant associations with response to NAC have been reported [19,21,22,27,28,29,30,31,32].

Currently, no universally approved biomarker is available to predict the response to NAC. Novel biomarkers at baseline that enable the identification of good and poor responders would be critical for therapeutic decision making for TNBC patients [33]. Nowadays, the literature regarding the tumor microenvironment is growing exponentially since this has been recognized as a regulator of carcinogenesis as well as immune evasion [34]. However, using immune parameters in daily clinical practice has not yet been generally implemented [19].

The goal of this review was to provide a systematic overview of the existing literature regarding baseline biomarkers present in cancer cells, the tumor microenvironment or peripheral blood of TNBC patients and their potential for predicting the pCR rate.

## 2. Methods

### 2.1. Literature Search

The databases Embase, MEDLINE and Web of Science were searched from 2010 to August 2022 for articles with the use of the controlled terms TNBC and NAC (Supplementary file S1). Only papers written in English were considered for inclusion. Reviews were excluded. The authors N.S. van den Ende and A.H. Nguyen independently reviewed the titles and abstracts of the identified articles. The full text of potentially relevant articles was evaluated independently by both N.S. van den Ende and A.H Nguyen. Disagreement was solved by discussion and reaching consensus.

### 2.2. Inclusion and Exclusion

**Figure 1:**
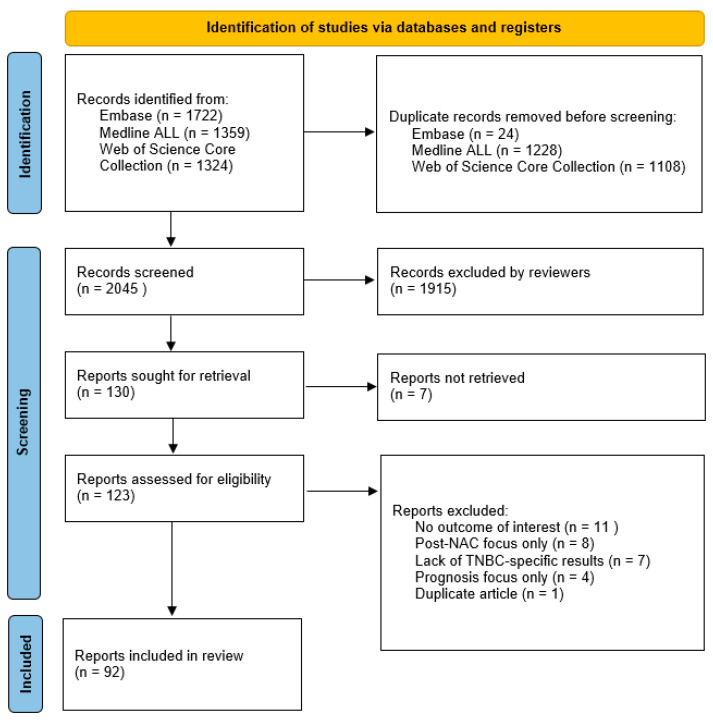
Flow diagram of inclusion and exclusion of papers for this review. NAC = neoadjuvant chemotherapy; TNBC = triple-negative breast cancer.

To be included in this review, the articles had to meet the following criteria: patients had to have primary invasive breast cancer without distant metastases and patients must have received NAC. A total of 2045 papers were screened and the papers that focused on the prediction of a pCR were of interest. The final exclusion of papers was based on the following criteria: no results for the outcome of interest, post-NAC focus only, lack of TNBC-specific results and focus on prognosis only. One duplicate article was discovered. In total, 92 papers were selected, revised and summarized for this article. Figure 1 illustrates the method of article selection.

### 2.3. Synthesis Method

Following the Preferred Reporting Items for the Systematic Reviews and Meta-analyses (PRISMA) 2020 statement, this review was written and subdivided into 4 parts: proteomic (divided into 2 sections: tumor cells and tumor microenvironment), genomic and transcriptomic features [35]. The PRISMA 2020 checklist can be found in Supplementary File S2.

## 3. Results

### 3.1. Proteomic Profile of Tumor Cells

Protein expression determined by immunohistochemistry is the method that is used mostly in daily diagnostics for tumor subtyping, because it is quick and relatively cheap. Many protein markers at baseline have been analyzed for their ability to predict the response rate to NAC.

TNBC is a highly proliferative breast cancer subtype, often associated with high Ki-67 expression [18]. The Ki-67 index is an indicator of proliferation activity and has been extensively investigated as a predictive marker for therapy response. Table 1 provides an overview of the literature regarding baseline Ki-67 protein levels and response to NAC in TNBC patients. In summary, various studies showed a statistically significant higher expression of Ki-67 in patients achieving a pCR versus patients without a pCR [20,25,32,36,37,38,39]. On the other hand, several other studies showed no statistically significant difference in Ki-67 expression levels between the pCR and non-pCR groups [19,24,26,40,41]. In addition, some studies reported that solely using Ki-67 expression is not sufficient to predict a pCR, suggesting that other markers are also needed for a robust prediction model [38,42,43]. Notably, it is important to consider that these studies used different cut-off values for what is considered a high Ki-67 protein expression (Table 1). All studies performed the Ki-67 staining on whole tissue slides, expect for the study by Kraus et al. that used tissue microarrays [41].

Another protein marker often investigated for its association with a pCR is the androgen receptor (AR). Approximately 65 to 85% of all TNBCs lack AR expression [21]. Multiple studies found that TNBC with a negative AR is correlated with a higher pCR rate than the TNBC AR+ subtype, indicating that the presence of AR decreases the chance of achieving a pCR [21,22,44]. There is a possibility that the lower pCR rate for AR-expressing tumors could be caused by a lower proliferation rate, which could make this subgroup more chemo-resistant. Mohammed et al. described that TNBC AR+ tumors showed a lower proliferation rate compared to TNBC AR- tumors [21]. In contrast, two other studies did not find an association between the AR status and the pCR rate [25,45]. Lately, it has also been suggested that the AR is involved in tumor cell immune evasion, resulting in a lower immune response and thus a lower chance of achieving a pCR [46].

The HER2 protein is a member of the *ERBB* ontology family [47]. Signaling through this family of receptors promotes cell proliferation and prevents apoptosis. TNBC lacks overexpression of the HER2 protein and HER2 negativity is classified, through immunohistochemistry, as score 0, score 1+ or score 2+ with negative in situ hybridization. Gluz et al. reported that a higher HER2 score (1+ or 2+ versus 0) was unfavorable for the pCR rate (*p* = 0.03) [38]. However, this result was dependent on the type of NAC and the association was only found in a subgroup treated with a nab-paclitaxel/carboplatin and not in the subgroup receiving nab-paclitaxel/gemcitabine. In a study by Denkert et al., there was no statistically significant difference in pCR rate between HER2-low cases (immunohistochemistry score of 1+ or 2+ without amplification) versus HER2-0 cases within the hormone receptor negative cohort [48]. Recently, there has been increased interest in revising the HER2 classification and implementing a HER2-low category, since these patients could benefit from novel therapeutic agents [49,50]. Moreover, this HER2-low subgroup has been reported to have a lower immune response, which is in concordance with the lower pCR rate reported by Gluz et al. [38,49].

The most common genetic mutation in TNBC is in the *TP53* gene, with a frequency of 84% [51,52]. The tumor suppressor protein p53 is involved in DNA repair mechanisms. Masuda et al. reported that tumor cells with a non-functional p53, determined by immunohistochemistry, do not respond to systemic therapy, due to a failure in apoptosis [25]. Several studies investigated the possible relation between p53 and a pCR. Most of these studies showed no significant association between immunohistochemical p53 expression or *TP53* mutation status and the response to NAC [20,25,26,53,54]. However, two studies did find a correlation between overexpression of p53 and a high pCR rate [19,43].

EGFR is a transmembrane glycoprotein, which is a member of the *ErbB* family of receptor tyrosine kinases [55]. It plays a role in cell proliferation, cell motility, tissue invasion, cell survival and angiogenesis [56]. Tang et al. reported that, within 40 TNBC patients, the overexpression of EGFR was significantly associated with a high pCR rate [55]. Another study reported no association between EGFR and a pCR [41]. Furthermore, Abdelrahman et al. analyzed the predictive impact of EGFR and reported that high EGFR expression was a negative predictor for achieving a pCR [56]. These contradictory results indicate that EGFR is not a robust marker for predicting the pCR rate.

For VEGFR2, vimentin and HAGE, a higher expression of these individual markers was associated with a higher likelihood of achieving a pCR [20,57,58]. VEGFR2 is a signaling protein involved in angiogenesis, vimentin is a cytoskeletal component of mesenchymal cells and HAGE is a helicase antigen. Various other biomarkers including FGFR4, NUP98, E-cadherin, Bcl2, ALDH1, tumor-associated stromal clusterin, TOPK, YAP1 and MMP7 were also correlated with the pCR rate [38,41,59,60,61,62,63,64]. A high expression of these markers was associated with an unfavorable response to NAC. It is important to note that the study that reported tumor-associated stromal clusterin in relation to therapy response also considered patients with a limited amount of residual tumor (residual cancer burden (RCB) score 1) as good responders [62]. FGFR4 is a fibroblast growth factor receptor and NUP98 is involved in nuclear import and export [38,59]. Notably, the association for FGFR4 was only found in the subgroup treated with a nab-paclitaxel/gemcitabine regimen and not in the subgroup treated with nab-paclitaxel/carboplatin [38]. In a subsequent validation set for E-cadherin, a tumor suppressor protein, no difference in expression level was found between good and poor responders [41,65]. Therefore, it remains unclear whether reduced E-cadherin staining can predict a pCR. Moreover, three other studies found no significant association between Bcl2 expression and the pCR rate [19,20,40]. ALDH1 is a marker of breast cancer stem cells, which are multipotent cells that are able to renew themselves [61]. TOPK plays a role in the growth of breast cancer cells, cell migration and invasion [63]. YAP1 is a protein that promotes transcription and MMP7 regulates and supports tumor proliferation and the inhibition of apoptosis [66,67].

Finally, for a number of other tumor-related biomarkers, such as GCS, CYP1A1, SOX10, GATA3, NF-κB, p63, CK5, CK5/6, CK14 and CK17, no association was found across several studies between expression levels and pCR rate [26,33,40,41,56].

### 3.2. Proteomic Profile of Tumor-Associated Immune Cells

The tumor microenvironment is of great importance for the survival, growth and metastasis of breast cancer cells [68]. Several immune parameters from the tumor microenvironment or from the circulation have been shown to have predictive and/or prognostic value.

Tumor-infiltrating lymphocytes (TILs) are lymphocytes that are present in and around the invasive tumor and they play a role in the tumor microenvironment where they can mediate adaptive immune responses against the tumor [69,70]. Overall, CD8+ T cells, CD4+ T cells and B cells are seen as TIL components. The scoring of TILs can be performed on H&E slides based on international guidelines by the International Immuno-Oncology Working Group for TIL assessment [71]. This working group has shown that TIL scores are reproducible with a high concordance among pathologists. Furthermore, TILs have also been reported to be prognostic markers, especially in TNBC, where a high density of TILs is associated with a survival benefit [72,73,74,75]. A high abundance of TILs has robustly been associated with a greater likelihood of achieving a pCR and a lower RCB score post-treatment in multiple studies [26,34,54,58,64,76,77,78,79,80,81,82,83,84,85,86,87,88] (Table 2). It is important to note, however, that in these studies, different cut-off values were used for TIL assessment, varying from ≥20% to ≥40%, ≥50% and even ≥60%. The RCB is a standardized approach to evaluate the pathologic response to chemotherapy, ranging from RCB-0 (equal to a pCR) to RCB-III (no response) [89,90]. In addition to the density of TILs, the phenotype of TILs and the location are also seen as important factors for the predictive value of TILs [91]. Most of the studies reported in this review did mention whether they analyzed stromal TILs or intratumoral TILs. However, there was a lack of mentioning whether the tumors have an immune-desert, immune-excluded or immune-inflamed appearance [91,92]. Zhang et al. also proposed TIL volume (TILV = % stroma in tumor x % stromal TILs) as a predictor for a pCR and showed that high TILV (≥1600) was associated with a pCR [34].

Zooming in on TILs, specifically high levels of CD8+ cells and a high CD8/CD4 ratio were reported to be associated with achieving a pCR [37,54,64,82,93,94,95]. These studies used immunohistochemical staining on whole tissue slides. High intratumoral and stromal CD4+ T cell density was also shown to be a significant predictor for a pCR, independent of the treatment [95,96]. T regulator cells (Tregs) are a subset of CD4+ T cells that keep the function of CD8+ T cells in-check [84]. The transcription factor FOXP3 is known for its involvement in suppressing CD8+ T cell immunity. A study by Abdelrahman et al. showed that the absence of FOXP3+ Tregs is associated with a pCR, likely as a consequence of an adaptive response in which the numbers and function of Tregs increase following an initial anti-TNBC CD8+ T cell response [84,97]. In agreement, Miyashita et al. found a higher pCR rate in patients with a high CD8+/FOXP3+ ratio compared to a low CD8+/FOXP3+ ratio [37]. However, the FOXP3 marker individually was not found to be associated with the pCR rate.

Recent reports have indicated that programmed cell death protein ligand 1 (PD-L1) expression can be used as a predictive marker of pCR rate [76,93,98,99,100]. Different PD-L1 antibodies with different scoring methods were used (SP142 Ventana, based on an immune cell score only, and 22C3 and SP263, based on a combined score of tumor cells and immune cells). Based on a cut-off of ≥1% on immunohistochemical staining for any of these three antibodies, between 50 and 80% of all TNBC tumors were PD-L1 positive and PD-L1 is known to be involved in the mechanism of immune escape and correlates with the overall immune cell infiltration [11,12,76]. Multiple studies indicated that a higher PD-L1 expression, for SP142, 22C3 and SP263, is associated with a higher likelihood of achieving a pCR [38,54,93,99,100]. In the study of Gluz et al., this association was only found in the carboplatin-treated group. The additional value of immunotherapy, combined with chemotherapy, in combination with the PD-L1 marker and pCR rate has been investigated recently. Mittendorf et al. reported that chemotherapy combined with pembrolizumab was beneficial for both PD-L1 positive and negative patients [12], whereas in the study by Schmid et al., the effect of the PD-L1 inhibitor was only seen in the PD-L1 positive subgroup [11]. Other studies have reported more contradictory results, which could partly be the result of the variation in PD-L1 assays, as reported by Savas et al. [70]. For example, Abdelrahman et al. found an association between negative PD-L1 status and achieving a pCR [84]. Studies by Foldi et al. and Ghosh et al. both found no association between PD-L1 expression and a pCR [76,98]. It has been reported that on a biologic level, PD-L1 expression is a marker for activated CD8+ TILs, indicating that elevated levels are correlated to an enhanced immune response [101], whereas on the therapeutic level, T cells that were originally inhibited by PD-L1 would reactivate again after a PD-L1 blockade, inducing an anti-tumor immune response.

CD73 plays a role in the catabolism of extracellular ATP to adenosine and is upregulated in regulatory T-cells in response to adenosine signaling [83]. It is highly expressed on mesenchymal stromal cells and infiltrating immune cells. Cerbelli et al. reported that low levels of CD73 expression on tumor cells correlate with an increased pCR rate. Another study by Cerbelli et al. analyzed whether a combination of TILs, PD-L1 and CD73 could better predict the pCR rate in 60 patients [31]. Twenty of the cases were TILs ≥ 50%, PD-L1 ≥ 1% and CD73 ≤ 40%, and after multivariate analysis these patients showed a significantly higher rate of response compared to the patients not reaching these cut-offs. Furthermore, the combination of these three markers was better at predicting a pCR than the individual biomarkers. Collectively, these results suggest that a combination of immune markers enables a more accurate prediction of a pCR.

Tumor-associated macrophages (TAMs) play an important role in tumor growth, angiogenesis, metastasis and treatment resistance. A high infiltration of CD163+ TAMs has been correlated with lower pCR rates after NAC [30,93]. In addition, Ye et al. found that a high infiltration of TAMs was associated with aggressive behavior (advanced stage, nodal metastasis, lymphovascular invasion) and poor prognosis [30]. Yam et al. assessed T cell receptor (TCR) clonality and found higher TCR clonality in TNBC patients who experienced a pCR [99]. They further reported that the ratio of CD8+ T cells and CD68+ TAMs in pre-treatment biopsies was higher in the NAC-sensitive group versus the NAC-resistant group (defined as RCB I-III). These findings suggest that a relative overabundance of T cells compared to TAMs in TNBC is associated with a pCR. Notably, spatial analysis showed that tumor cells in NAC-sensitive tumors were in closer proximity to CD8+ T cells compared to NAC-resistant tumors. Furthermore, it was found that NAC-sensitive tumors had a higher TCR clonality compared to NAC-resistant tumors, and this clonal expansion of TILs, particularly the CD8+ T cells, is suggestive of anti-tumor reactivity, based on the NAC response.

Furthermore, recent research suggests that Ki-67 expressed on immune cells could also be important for immune oncology benefits (Bianchini et al. [102]).

Besides lymphocytes and macrophages, other immune cell subpopulations show aberrant abundance in TNBC. Neutrophils are known to promote tumor cell proliferation, angiogenesis and distant metastasis [103]. Normally, the neutrophil-to-lymphocyte ratio (NLR) is low in tumors with high lymphocyte activity, which is often found in TNBC [29]. Chemotherapy could activate innate and adaptive immune cells and particularly evoke immune responses in patients with a low NLR. Neutrophil counts in tumor tissue and intratumoral NLR did not show a significant difference between the pCR and the non-pCR group [29,104]. Interestingly, in the study by Tokumaru et al., different regimens in five independent cohorts were studied, which all showed that high intratumoral NLR levels were associated with worse prognosis.

### 3.3. Immune Cells in Peripheral Blood

Frequencies of circulating immune cells are also linked to the response to NAC. The examination of circulating immune cells is simple, less invasive and less expensive compared to obtaining tissue-based immune parameters. A significant association was reported between a low NLR in circulating blood and the ability to achieve a pCR in several studies [104,105,106,107,108]. However, other studies did not find a significant association between the pCR rate and NLR levels [77,98,104,109]. Low hemoglobin levels, albumin levels, lymphocyte counts and platelet counts (HALPs) and a high platelet-to-lymphocyte ratio were also reported as markers of a poor efficacy of NAC [108]. However, multiple other studies found no significant association between a pCR or distant recurrence rate and absolute or relative baseline blood cell counts of neutrophils, eosinophils, lymphocytes, monocytes, platelets and the platelet-to-lymphocyte ratio [77,98,110]. Currently, markers in peripheral blood are not robust enough to solely predict the pCR rate.

### 3.4. Genomic Profile

Genetic mutations in the DNA of the tumor cell have extensively been analyzed for their role in the response rate.

Besides the common *TP53* mutation, other genes are also often dysregulated in breast cancer. For example, the *PIK3CA* gene is mutated in 16% of primary TNBCs and mutations in *PIK3CA* reduce the dependency of tumor cells on growth factors, promoting cell growth and transformation [111]. Research thus far suggests that patients with a *PIK3CA*-enhancing mutation are less likely to achieve a pCR compared to *PIK3CA* wild-type tumors, indicating that PI3K inhibitors could possibly enhance chemotherapeutic sensitivity [28,111,112]. However, this association has shown to be dependent on the specific type of *PIK3CA* mutation and NAC regimen.

On average, 10% to 15% of all TNBC patients carry a *BRCA1* or *BRCA2* germline mutation and *BRCA1* mutation carriers are more likely to develop TNBC [15,113]. BRCA1/BRCA2 proteins play an important role in DNA repair via homologous recombination. Therefore, these mutations generally lead to a homologous recombination deficiency. Currently, most TNBC patients, *BRCA* mutation carriers or not, are treated with NAC. The pCR rate has been reported to be between 35% and 70% for TNBC patients with a *BRCA* mutation, receiving the standard NAC regimen [114,115]. Due to the mutation in the DNA damage repair mechanism, it could be possible that these patients show a difference in response to treatment. Indeed, two studies reported that patients with a germline *BRCA1* mutation achieved a higher pCR rate than patients without the *BRCA* mutation [15,116]. However, in one study, this was only significant in the anthracycline (with or without taxane) regimen and not in the taxane-based regimen where it showed a similar pCR rate between germline *BRCA1* mutation carriers and non-carriers. However, several other studies did not find a statistically significant difference between the pCR rates of good versus poor responders based on the germline or somatic *BRCA1/2* mutation status [23,114,117,118]. This is possibly due to a small sample size of the mutation carriers or differences in chemotherapy regimen.

Watanabe et al. found that on the epigenetic DNA level, *BRCA1* methylation levels are higher in patients with a pCR than those without a pCR [119]. Germline mutations of homologous recombination genes such as *ATM* are involved in breast cancer susceptibility. It was found that there were lower levels of DNA methylation in the *ATM* gene in cases with a pCR than in cases with a poor response. Another study by Meyer et al. reported nine differentially methylated regions to be associated with the response to NAC [120].

Besides the *BRCA* mutation status, homologous recombination deficiency itself has also been associated with pCR rates. This group of tumors is also called BRCAness or BRCA-like. These sporadic cancers share phenotypic overlap with *BRCA1/2* germline-mutated tumors. However, these patients lack a detectable germline mutation but do have a homologous recombination error. In contrast to the positive predictive value of the *BRCA* mutation status, Akashi-Tanaka et al. reported that those with non-BRCAness tumors achieved a pCR more often than those with BRCAness tumors [121]. On the contrary, Telli et al. mentioned that homologous recombination-deficient patients are associated with achieving a pCR more often than patients without homologous recombination deficiency [117]. Moreover, also within wild-type *BRCA* patients, homologous recombination deficiency was correlated with a higher pCR rate. A study by Huang et al. found that the mutation status of 10 DNA repair genes involved in homologous recombination could predict the response to NAC [114]. Tumors with a positive mutation status for such genes would achieve a pCR more often than tumors with a negative mutation status.

Topoisomerase IIa (*TOP2A*) is a nuclear DNA-binding enzyme which is critical for obtaining a relaxed DNA structure, important for replication [122]. Data regarding *TOP2A* are inconsistent. No significant correlation between *TOP2A* overexpression and response rate to NAC was reported by Sakuma et al. [43]. The study of Loibl et al. found that tumors with amplification of *TOP2A* showed a decreased pCR rate compared with tumors without amplification of *TOP2A* [112]. In contrast, Rao et al. described that patients with high *TOP2A* expression were more likely to achieve a pCR [79].

### 3.5. Transcriptomic Profile

Some studies developed a gene signature consisting of coding genes and/or long non-coding RNAs (lncRNA), to predict the response to NAC. It is noteworthy that half of the genome is transcribed through lncRNAs, which are RNA transcripts of more than 200 nucleotides without coding capacity. Normally, lncRNAs are regulatory molecules that have been associated with the pathology of breast cancer. A study by Wang et al. investigated whether a specific RNA signature, based on lncRNA and coding gene expression, could predict the pCR rate and demonstrated that a response score signature consisting of one lncRNA (*BPESC1*) and two coding genes (*WDR72* and *GADD45A*) could significantly distinguish between patients achieving a pCR and non-pCR [123]. When the response score was higher, patients were more likely to achieve a pCR. *GADD45A* is involved in growth arrest and DNA damage pathways [124]. Zheng et al. found that a gene signature of three coding genes (*TCF3*, *CREB1* and *CEP44*) and two lncRNAs (NR 023392.1 and NR 048561.1) could predict the pCR to NAC [125]. *TCF3* is part of the Wnt pathway-associated TCF/LEF transcription factor family and *TCF3* is upregulated in cancers where it promotes proliferation and metastasis. *CREB1* is a DNA-binding protein which stimulates transcription and it is also involved in tumor proliferation and metastasis [125,126].

Multiple other gene signatures, scores and (random forest) models investigated in different studies have also been reported to be predictive for pCR rate [39,95,127]. Multiple favorable gene signatures are correlated with an immunogenic tumor microenvironment, further indicating the essential role of an immune-active tumor microenvironment for achieving a pCR.

Furthermore, higher expression levels of *TMBS15A, SIRT5, SPARC, LAG-3, SFRP1, CCND1, SCD5, ILF2, IDO1, CTLA4, NFKB1, MAPK1, TRAF1, CXCL13, CXCL16, GZMK, IL7R, CR2, CD19, MS4A1, IL33* and *MELK* are associated with a higher likelihood of achieving a pCR [8,29,54,64,94,128,129,130,131,132]. Many of these genes, such as *LAG-3, IDO1, CTLA4, CXCL13, CXCL16, GZMK, IL7R, CD19* and *IL33* have reported immune functions, and their putative relation with a pCR is in line with findings on the immune profile, as discussed above. Patients with low expression levels of *ITPKC* and *HO-1* and a lack of *PCDH17* methylation showed higher pCR rates [18,27,32]. Furthermore, an increased expression of the neutrophil-associated genes *DEFB1*, *DEFB103A*, *DEFB4A* and *FCAR* was seen in patients who failed to achieve a pCR [29]. These findings are in concordance with the previously reported results about the negative association between a high NLR and achieving a pCR [104,105,106,107,108]. Gene expression profiles have been reported to be of importance for therapy responses by Lehmann et al. [5]. It was reported that there are six TNBC subtypes, based on gene expression profiles, and that these subtypes not only have a distinct phenotype but also have variable chemo-sensitivity and pCR rate [5,95]. Echavarria et al. reported that the pCR rate was significantly different between the different TNBC subtypes (Echavarria et al. [133]). The basal-like subtype has been associated with the highest pCR rate and is characterized by the high expression of DNA damage response genes and a high mRNA expression of Ki-67 [5]. Moreover, the immunomodulatory subtype is characterized by genes involved in immune cell processes, of which many have been reported earlier in this review, and is associated with a favorable prognosis. Furthermore, an immune gene signature of the combination *IDO1*, *LAG3*, *STAT1* and *GZMB* did not show an association with pCR rate [134].

MicroRNAs (miRNAs) are considered to influence the development and progression of breast cancer and are small non-coding RNAs of 25 nucleotides in length. Gene expression can be negatively regulated by miRNAs by inhibiting the translation or degradation of the target messenger RNA (mRNA). Lately, the role of several microRNAs in the chemo-resistance of TNBC tumors and their predictive value for the rate of response to NAC has been studied extensively. The miR-143-5p, miR-30a, miR-9-3p, miR-770 and miR-18a showed differential expressions between good and poor responders to NAC [36,135,136]. MiR-143-5p was significantly less expressed in patients who reached a pCR compared with patients who did not reach a pCR [136]. A high expression of miR-18a was shown in patients that were chemo-resistant to paclitaxel [135]. Some of these discriminatory miRNAs were deregulated in patients achieving a pCR, indicating that alterations in the miRNAs could be involved in the achievement of a pCR through the stimulation of particular oncogenic signaling pathways such as TGFB, PI3K/AKT, ErbB, VEGF/MAPK, FOXO, FAK, JAK/STAT and mTOR [136]. Not all miRNAs are associated with the response rate to NAC. Kolacinska et al. analyzed different miRNAs and identified that 3 out of 19 miRNAs, miR-200b-3p, miR-190a and miR-512-5p, could distinguish patients with a pCR from patients not achieving a pCR based on expression levels. They also observed that higher miR-200b-3p, higher miR-190a and lower miR-512-5p expression levels showed a trend towards reaching a higher pCR rate, but there was no statistically significant difference compared to the non-pCR group [137].

Exosomes are small membranous vesicles of around 30 to 100 nanometers that can contain lipids, proteins or miRNAs secreted by cells into the blood [138]. Exosomal miRNA can transmit information to the surrounding microenvironment. Sueta et al. reported that 16 exosomal miRNAs were expressed significantly differently between patients achieving a pCR and non-pCR. A signature of four of these upregulated miRNAs, miR-4448, miR-2392, miR-2467-3p and miR-4800-3p, was the most optimal to distinguish between patients with and without a pCR.

## 4. Discussion

**Figure 2:**
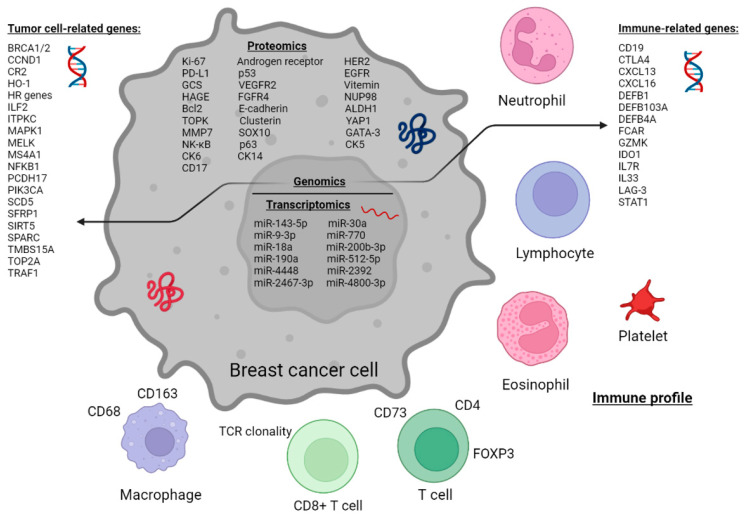
Schematic representation of putative predictive markers linked to pCR after NAC in TNBC patients.

This review focused on comprehensively charting and describing biomarkers at baseline that could predict a pCR following NAC treatment for TNBC, on proteomic, genomic and transcriptomic levels. Various biomarkers have been investigated for their correlation with pCR rate (Figure 2). Despite the large number of studies investigating which biomarkers are associated with a pCR, no ideal marker has reached the robustness and confidence to become widely implemented in clinical practice.

Based on this review, it has become clear that the immune microenvironment is critical for the response to NAC. The higher density of TILs, particularly CD8+ T cells, has been shown to be associated with achieving a pCR, indicating the predictive value of TILs as reported in a total number of 18 articles. The favorable involvement of CD4+ T regulatory cells and the unfavorable involvement of CD163+ TAMs and CD73+ tumor cells were also presented to correlate with the pCR rate. Thus, the pre-treatment activation of the CD8+ T cells against TNBC or their control via adaptive feedback mechanisms (such as evidenced by Tregs and TAMs) likely positively influences the response to NAC. The presence of CD8+ T cells is known to induce apoptosis of cancer cells, and CD4+ T cells are known for their helping function in maintaining the anti-tumor role of CD8 [139]. Chemotherapy can alter the microenvironment and with higher numbers of CD8+ and CD4+ T cells at baseline, these responses could be further enhanced. Indeed, a study by Emens et al. has shown that cyclophosphamide can assist immune cells to develop and mature, leading to better responses to treatment [140]. Furthermore, Voorwerk et al. reported in preclinical research that chemotherapy renders immunomodulatory properties through the upregulation of genes associated with T cell cytotoxicity, inflammation and PD-L1 pathways [141]. PD-L1 has also been reported as a predictive marker, but results were contradictory, which could partially be due to the variations in PD-L1 antibodies. CD163+ TAMs have an M2-like macrophage phenotype and CD73 is an enzyme that produces immunosuppressive extracellular adenosine [93,142,143]. A study by Jinushi et al. suggested that CD163+ TAMs can produce milk-fat globule epidermal growth factor-8, which in turn can mediate drug resistance through STAT3 and hedgehog signals [144]. For the other immune markers, such as eosinophil count, the platelet-to-lymphocyte ratio and NLR, results varied, and no clear association was found.

On the tumor cell level, Ki-67 and AR seem to be fairly robust predictive markers of a pCR. Nevertheless, for Ki-67, the results reported are somewhat contradictory, but there were more studies (n = 7) that found an association between a high Ki-67 expression and pCR rate compared to the studies that did not find a significant association (n = 5). In addition, the studies reporting an association were based on larger cohorts compared to the studies without an association. On the genomic level, *PIK3CA* seems to be the only biomarker that has shown an association with a pCR in multiple articles. Furthermore, various biomarkers have been found to be associated with a pCR but have only been reported infrequently. For these biomarkers, additional research is needed to validate the association with a pCR.

To our knowledge, this is the first systematic review giving an exhaustive overview of all reported biomarkers of a pCR in NAC-treated TNBC at the proteomic, genomic and transcriptomic levels. This review was written as a narrative (qualitative) approach aiming to achieve a better understanding of the potential biological effects of individual markers on the pCR rate. A quantitative approach meta-analysis was not possible because of the heterogeneity of the articles in this search. The studies varied in biomarkers, patient populations, sample size, treatment regimen and other study settings, which makes it difficult to generalize the results found in these studies. Moreover, a lack of standardized threshold settings for immunohistochemical stains and scoring methods, and peripheral blood cell counts, increases the variety between the studies.

Most of the studies included in this review followed the ASCO/CAP guidelines for ER and PR scoring, using a cut-off of <1%. However, nine studies used a cut-off of <10% for an ER negative status [24,25,43,55,77,106,119,122,130]. Four studies used the Allred score to determine ER status and one study used the H-score [26,41,61,78,81]. The definition of a pCR also differed across the studies included in this review. Mainly, a pCR was defined as being where no invasive residual tumor cells were present in the breast and axillary lymph nodes [145]. Other used definitions were the absence of invasive and in situ carcinoma in the breast and axillary lymph nodes after completing the NAC regimen, and in one study an RCB1 score was seen as a pCR [62,90]. However, the definitions are very comparable, so it is unlikely that the difference in pCR definition affected the outcome of results.

Currently, treatment decisions are based on baseline prognostic clinicopathologic features, so the additional value of predictive biomarkers should be studied on top of this. Given the rapid developments in the field of neoadjuvant treatment of TNBC patients, including the addition of neoadjuvant pembrolizumab, it becomes even more important to select those patients that could be treated with chemotherapy alone, to keep the health care system affordable. In addition, TIL-high TNBC patients have been reported to have a good prognosis without any systemic treatment [74]. Therefore, there is a need for a reproducible predictive biomarker to be used on top of the baseline prognostic features.

A clinical useful predictive biomarker is analytically valid, reproducible and able to select patients with a high or low chance of a pCR after NAC (for example >90% or <10%) [146,147,148]. As described in this review, there is plenty of literature reporting significant associations between biomarkers and therapy response, mainly the TILs and the expression of Ki-67. Based on the literature, the abundance of TILs is the most robust biomarker so far. Since both TILs and Ki-67 are relatively easy to implement in daily clinical practice, it could be considered that Ki-67 be reported as well as the density and location of TILs. However, the pCR rate varies substantially across studies in TIL-high and Ki-67-high cases, so the predictive value of these markers by themselves does not seem to be sufficiently strong enough yet to guide clinical decision making. Artificial intelligence techniques, such as deep learning, are being developed to assess the H&E slides for the density of TILs and spatial information [149]. Future research on early readouts for therapy response, during the NAC period, could also contribute to the optimization of the treatment schedule.

## 5. Conclusions

In conclusion, several markers have been associated with the pCR rate, with immune markers being the most promising. However, several other markers that relate to cancer cells, the tumor microenvironment and the peripheral blood also affect chemo-sensitivity. The combination of tumor cell and immune markers, such as Ki-67 expression and TIL density, in a prediction model is likely to yield the best stratification of TNBC patients with respect to response to NAC.

## Figures and Tables

**Table 1:** Overview of articles regarding Ki-67 expression at baseline and the association with a pCR after NAC in TNBC patients.

Author	Year of Publication	Study Size	Cut-Off Value High vs. Low	*p*-Value of the Association between Ki-67 and pCR	General Result
Masuda et al. [25]	2011	N = 33	50%	*p* = 0.03	The percentage of patients with a pCR who had high Ki-67 expression was 50%, whereas 15% had low Ki-67 expression
Sakuma et al. [43]	2011	N = 44	50%	*p* > 0.9999	The percentage of patients with a high Ki-67 expression who had a pCR was 39%, compared to 36% of patients with a low Ki-67 expression
Kraus et al. [41]	2012	N = 56	25%	*p* = 0.542	Patients with a pCR had a mean Ki-67 expression of 61% (range 35–90%), compared to 58% (range 5–95%) in patients without a pCR
Kawate et al. [42]	2013	N = 205	14%	*p* = 0.1011	The percentage of patients with a high Ki-67 expression who had a pCR was 28%, compared to 16% of the patients with a low Ki-67 expression
Miyashita et al. [37]	2014	N = 110	57.5%	*p* = 0.002	High Ki-67 expression was associated with a pCR rate of 70%, while low Ki-67 expression was associated with a pCR rate of 5%
Kim et al. [19]	2015	N = 198	10%	*p* = 0.056	High Ki-67 expression was associated with a pCR rate of 19.5%, while low Ki-67 expression was associated with a pCR rate of 6%
Baba et al. [40]	2016	N = 34	35%	*p* = 0.66	In patients with a pCR, the mean Ki-67 expression was 55% ± 30 standard deviation. In patients without a pCR, the mean Ki-67 expression was 80% ± 12 standard deviation for partial response, 73% ± 8 standard deviation for stable disease and 55% ± 41 standard deviation for progressive disease
Garcia-Vazquez et al. [36]	2017	N = 18	Mean expression of Ki-67	64% expression of Ki-67 in pCR vs. 51% expression of Ki-67 in non-pCR	In patients with a pCR, the median Ki-67 expression was 71% (range 30–90), compared to 62% in the non-pCR group (range 15–90)
Bignon et al. [24]	2018	N = 53	Mean pCR vs. non-pCR	*p* = 0.48	The mean Ki-67 expression in the pCR group was 68%, compared to 64% in the non-pCR group
Guestini et al. [20]	2019	N = 148	53%	*p* = 0.023	Patients with a pCR had higher expression levels of Ki-67
Gluz et al. [38]	2020	N = 336	Not mentioned	*p* < 0.001	n/a
Kong et al. [32]	2020	N = 280	20%	*p* = 0.451	n/a
Van Bockstal et al. [26]	2020	N = 35	20%	*p* = 1.000	In the pCR group, no patients had a low Ki-67 expression. In the non-pCR group, 4% had low Ki-67 expression, while 96% had high Ki-67 expression
Zuo et al. [39]	2022	N = 127	40%	*p* = 0.028	n/a

**Table 2:** Overview of articles regarding density of TILs at baseline and the association with a pCR after NAC in TNBC patients.

Author	Year of Publication	Study Size	Cut-Off Value High vs. Low	Location of TILs: Stromal (s) or Tumoral	*p*-Value of the Association between pCR and Density of TILs	General Result
Ono et al. [81]	2012	N = 102	50%	sTILs	*p* = 0.05	TIL-high patients had a pCR rate of 37%, compared to 16% in TIL-low patients
Abdel-Fatah et al. [58]	2016	N = 110	60%	sTILs and intratumoral TILs	*p* = 0.005	TIL-high patients had a pCR rate of 53%, compared to 15% in TIL-low patients
Castaneda et al. [82]	2016	N = 98	Continuous scale (per 10% increment)	sTILs	*p* = 0.0251	A higher median TIL percentage of 40% ± 17.5 interquartile deviation was associated with a pCR, compared to a median of 30% ± 20 interquartile deviation TIL percentage in the non-pCR patients. When a cut-off of 50% was used for TIL-high vs. TIL-low, no significant association was found for pCR rate (*p* = 0.16)
Rao et al. [79]	2017	N = 52	35% (CD4) and 15% (CD8)	sTILs	*p* = 0.004 (CD4+ TILs) and *p* = 0.006 (CD8+ TILs)	When CD4+ TILs were high, 41.9% achieved a pCR, compared to 4.8% in cases with low CD4+ TILs. When CD8+ TIL levels were high, 47.6% achieved a pCR, compared to 12.9% of cases with low CD8+ TIL levels. Patients with both high CD4+ and CD8+ TIL levels had a pCR rate of 71.4%
Herrero-Vicent et al. [80]	2017	N = 164	40%	sTILs	*p* = 0.001	TIL-high cases had a pCR rate of 88%, compared to 9% of the TIL-low cases
Würfel et al. [85]	2018	N = 146	50%	sTILs	*p* < 0.01	TIL-high cases had a pCR rate of 67%, compared to 33% of the TIL-low cases
Ruan et al. [86]	2018	N = 166	20% for sTIL and 10% for intratumoral TILs	sTILs and intratumoral TILs	*p* = 0.006 (stromal) and *p* = 0.04 (intratumoral)	n/a
Zhang et al. [34]	2018	N = 58	60%	sTILs	*p* = 0.01	The percentage of the pCR cases that were TIL-high was 46%, compared to 16% in the non-pCR group
Ochi et al. [78]	2019	N = 80	9%	sTILs	*p* < 0.001	TIL-high cases had a pCR rate of 44%, compared to 4% of TIL-low cases
Van Bockstal et al. [26]	2020	N = 35	40%	sTILs	*p* = 0.002 (2-tier) *p* = 0.013 (continuous percentage)	The percentage of the pCR cases that were TIL-high was 62%, compared to 9% in the non-pCR group
Cerbelli et al. [83]	2020	N = 75	Low: ≤9% Intermediate: ≥10–49% High: ≥50%	sTILs	*p* = 0.037	TIL-high cases had a pCR rate of 76.5%, compared to 16% in the TIL-intermediate cases and 42% in the TIL-low cases
Foldi et al. [76]	2021	N = 69	Low: ≤9% Intermediate: ≥10–29% High: ≥30%	sTILs	*p* = 0.0167	TIL-high cases has a pCR rate of 57%, compared to 60% in the TIL-intermediate cases and 29% in the TIL-low cases
Abdelrahman et al. [84]	2021	N = 50	50%	sTILs	*p* < 0.02	TIL-high cases had a pCR rate of 71%, compared to 28% of TIL-low cases
Ademuyiwa et al. [54]	2021	N = 127	Continuous scale (per 10% increment)	sTILs	*p* = 0.05	n/a
Lusho et al. [77]	2021	N = 120	30%	Not mentioned	*p* = 0.007	TIL-high cases had a pCR rate of 54%, compared to 24% in TIL-low cases
Yuan et al. [64]	2021	N = 433	20%	sTILs	*p* = 0.014	n/a
Goda et al. [88]	2022	N = 20	50%	sTILs	*p* = 0.002	The percentage of the pCR cases that were TIL-high was 67%, and 18% of the non-pCR cases were TIL-high
Khoury et al. [87]	2022	N = 129	Mean pCR vs. non-pCR	sTILs	*p* = 0.0003	n/a

## Data Availability

Not applicable.

## Supplementary Materials

The supporting information can be downloaded at https://www.mdpi.com/article/10.3390/ijms24032969/s1.

## Abbreviations

ALDH1	Aldehyde dehydrogenase 1 family member A1
AR	Androgen receptor
ATM	Ataxia telangiectasia-mutated
ATP	Adenosine 5′-triphosphate
Bcl2	B-cell lymphoma 2
BPESC1	Blepharophimosis, epicanthus inversus and ptosis candidate 1
BRCA	Breast cancer
CCND1	Cyclin D1
CK	Cytokeratine
CD19	Cluster of differentiation 19
CEP44	Centrosomal protein 44
CR2	Complement component 3d receptor 2
CREB1	CAMP responsive element binding protein 1
CTLA4	Cluster of differentiation 152
CXCL	Chemokine (C-X-C motif) ligand
DEFB	Defensin beta
DFS	Disease-free survival
EGFR	Epidermal growth factor receptor
ER	Estrogen receptor
FCAR	Fc fragment of IgA receptor
FOXP3	Forkhead box P3
FGFR4	Fibroblast growth factor receptor 4
GADD45A	Growth arrest and DNA damage inducible alpha
GATA3	GATA binding protein 3
GCS	Glucosylceramide synthase
GZMB	Granzyme B
GZMK	Granzyme K
HAGE	Helicase antigen
HER2	Human epidermal growth factor 2
HO-1	Heme oxygenase 1
IDO1	Indoleamine 2,3-dioxygenase 1
IL7R	Interleukin-7 receptor
IL33	Interleukin 33
ILF2	Interleukin enhancer binding factor 2
ITPKC	Inositol-trisphosphate 3-kinase C
LAG-3	Lymphocyte activation gene-3
LncRNA	Long non-coding RNA
MAPK1	Mitogen-activated protein kinase 1
MELK	Maternal embryonic leucine zipper kinase
MFS	Metastasis-free survival
miRNA	MicroRNA
MMP7	Matrix metalloproteinase 7
MS4A1	Membrane spanning 4-domains A1
NAC	Neoadjuvant chemotherapy
NFKB1	Nuclear factor kappa B subunit 1
NUP98	Nucleoporin 98 and 96 precursor
OS	Overall survival
PCDH17	Protocadherin 17
pCR	Pathological complete response
PD-L1	Programmed cell death protein ligand 1
PIK3CA	Phosphatidylinositol-4,5-bisphosphate 3-kinase catalytic subunit alpha
PR	Progesterone receptor
RCB	Residual cancer burden
RFS	Recurrence free survival
SCD5	Stearoyl-CoA desaturase 5
SFRP1	Secreted frizzled related protein 1
SIRT5	Sirtuin 5
SPARC	Secreted protein acidic and rich in cysteine
SOX10	SRY-box transcription factor 10
STAT1	Signal transducer and activator of transcription 1
(s)TIL	(Stromal) tumor-infiltrating lymphocyte
TCF3	Transcription factor 3
TCR	T cell receptor
TMBS15A	Thymosin beta-15A
TRAF1	TNF receptor-associated factor 1
TILV	Tumor-infiltrating lymphocyte volume
TP53	Tumor protein 53
TOP2A	Topoisomerase IIa
TOPK	T-LAK-cell originated protein kinase
Tregs	T regulator cells
VEGFR2	Vascular endothelial growth factor receptor 2
WDR72	WD repeat domain 72
YAP1	Yes1-associated transcriptional regulator
