# Supplementary material for: Triple-Negative Breast Cancer and Predictive Markers of Response to Neoadjuvant Chemotherapy: A Systematic Review
Source: Int J Mol Sci. 2023 Feb 3;24(3):2969. doi: 10.3390/ijms24032969 (PMC9918290; doi:10.3390/ijms24032969)
Supplement: Supplementary file 1 [file ijms-24-02969-s001.zip › Supplementary file S1 TNBC predictive biomarkers pCR.pdf]

Supplementary file S1

Literature search in databases.

Literature search in databases Embase, Medline and Web of Science, The first search was performed on the 27th of august 2021 and an update of the literature search was performed on the 10th of august 2022. The same search method was used for the primary search and the update literature search.

Triple negative breast carcinoma neoadjuvant chemotherapy

**Database searched**	**via**	**Years of coverage**	**Records**	**Records after duplicates removed**
Embase	Embase.com	1971 - Present	1722	1698
Medline ALL	Ovid	1946 - Present	1359	131
Web of Science Core Collection*	Web of Knowledge	1975 - Present	1324	216
**Total**			**4405**	**2045**

*Science Citation Index Expanded (1975-present) ; Social Sciences Citation Index (1975-present) ; Arts & Humanities Citation Index (1975-present) ; Conference Proceedings Citation Index- Science (1990-present) ; Conference Proceedings Citation Index- Social Science & Humanities (1990-present) ; Emerging Sources Citation Index (2015-present)

Update literature search resulted in 387 new references

Embase.com

('breast tumor'/exp OR ((breast OR mammary) NEAR/3 (tumor* OR tumour* OR cancer* OR carcinoma* OR neoplas*)):Ab,ti) AND ('triple negative breast cancer'/exp OR ('estrogen receptor negative breast cancer'/de AND 'human epidermal growth factor receptor 2 negative breast cancer'/de AND 'progesterone receptor negative breast cancer'/de) OR ((triple NEAR/3 negative) OR tnbc):ab,ti) AND ('neoadjuvant chemotherapy'/exp OR 'neoadjuvant therapy'/de OR ((neoadjuvant* OR neo-adjuvant*)):Ab,ti) AND (histology/de OR histopathology/de OR 'pathological complete response'/de OR 'complete response'/de OR 'pathologic complete response'/de OR 'complete response rate'/de OR 'partial response'/de OR 'treatment response'/de OR (histolog* OR histopatholog* OR respon* OR pcr):Ab,ti) AND ('cohort analysis'/exp OR 'longitudinal study'/de OR 'prospective study'/de OR 'retrospective study'/de OR 'observational study'/de OR 'clinical trial'/exp OR 'major clinical study'/de OR 'patient characteristics'/de OR 'case study'/de OR patient/de OR (cohort* OR longitudinal* OR prospectiv* OR retrospectiv* OR observational* OR trial* OR cases OR (case* NEAR/3 stud*) OR patients):Ab,ti) NOT ('nuclear magnetic resonance imaging'/exp/mj OR ((magnetic NEAR/3 resonan*) OR mri OR mr-imag* OR ((patient* OR tumor* OR tumour*) NEAR/6 characteristic*)):ti) NOT [conference abstract]/lim NOT [conference review]/lim NOT ([animals]/lim NOT [humans]/lim) AND [english]/lim

Medline ALL Ovid

(exp Breast Neoplasms/ OR ((breast OR mammary) ADJ3 (tumor* OR tumour* OR cancer* OR carcinoma* OR neoplas*)).ab,ti.) AND (Triple Negative Breast Neoplasms/ OR ((triple ADJ3 negative) OR tnbc).ab,ti.) AND (Neoadjuvant Therapy/ OR neoadjuvant therapy/ OR ((neoadjuvant* OR neo-

adjuvant*)).ab,ti.) AND (Histology/ OR (histolog* OR histopatholog* OR respon* OR pcr).ab,ti.) AND (exp Cohort Studies/ OR Observational Study/ OR exp Clinical Trial/ OR Case Reports/ OR Patients/ OR (cohort* OR longitudinal* OR prospectiv* OR retrospectiv* OR observational* OR trial* OR cases OR (case* ADJ3 stud*) OR patients).ab,ti.) NOT (exp * Magnetic Resonance Imaging/ OR ((magnetic ADJ3 resonan*) OR mri OR mr-imag* OR ((patient* OR tumor* OR tumour*) ADJ6 characteristic*)).ti.) NOT (exp animals/ NOT humans/) AND english.la.

Web of Science

TS=((((breast OR mammary) NEAR/2 (tumor* OR tumour* OR cancer* OR carcinoma* OR neoplas*))) AND (((triple NEAR/2 negative) OR tnbc)) AND (((neoadjuvant* OR neo-adjuvant*))) AND ((histolog* OR histopatholog* OR respon* OR pcr)) AND ((cohort* OR longitudinal* OR prospectiv* OR retrospectiv* OR observational* OR trial* OR cases OR (case* NEAR/2 stud*) OR patients))) NOT TI=(((magnetic NEAR/2 resonan*) OR mri OR mr-imag* OR ((patient* OR tumor* OR tumour*) NEAR/5 characteristic*))) AND DT=(article) AND LA=(english)
